# Evaluation of Immune Characteristics and Factors Associated with Immune Response following Hepatitis B Vaccination among Ghanaian Adolescents

**DOI:** 10.1155/2024/9502939

**Published:** 2024-05-24

**Authors:** Samuel Asamoah Sakyi, Joseph Badu Gyapong, Ebenezer Krampah Aidoo, Alfred Effah, Simon Koffie, Oscar Simon Olympio Mensah, Isaac Arddey, Godwin Boakye, Stephen Opoku, Benjamin Amoani, Robert Amadu Ngala

**Affiliations:** ^1^Department of Molecular Medicine, School of Medicine and Dentistry, Kwame Nkrumah University of Science and Technology, Kumasi, Ghana; ^2^Department of Medical Laboratory Technology, Accra Technical University, Accra, Ghana; ^3^Department of Neurosurgery, Heersink School of Medicine, University of Alabama at Birmingham, Birmingham, Alabama, USA; ^4^Department of Biomedical Sciences, University of Cape Coast, Cape Coast, Ghana

## Abstract

**Background:**

WHO recommends HBV-negative babies in high-prevalence (8%) countries receive anti-HBV vaccination. Ghana initiated mass immunization in 2002, but concerns remain about vaccine effectiveness and long-term protection. We evaluated immune characteristics and factors following hepatitis B vaccination among Ghanaian adolescents who received HBV vaccines.

**Methods:**

In this longitudinal cross-sectional study, 74 participants were enrolled from the Kumasi Metropolis, Ghana. Sociodemographic and lifestyle characteristics of participants were obtained using a questionnaire. Blood samples were obtained before and after booster administration for anti-HBsAg, IL-6, and IL-10 estimations using ELISA kit (Shanghai Chemical Ltd., China). Anti-HBsAg titers ≥10 mIU/ml were considered protective. Statistical analyses were done using SPSS version 26.0 and R programming language, *p* < 0.05 was considered statistically significant.

**Results:**

We found 100% seroconversion rate, with 25.7% seroprotection rate (anti-HBsAg >10 mIU/ml). Gender (*p*=0.009), age (*p*=0.001), and exercising (*p*=0.044) were significantly associated with seroprotection. Following booster administration, 59.4% were hyporesponders (10 ≤ anti-HBsAg titre ≤99 mIU/ml) whilst 40.6% were good responders (anti-HBsAg titre ≥100 mIU/ml). Exercise (*p*=0.034) was significantly associated with immune response after booster administration. Moreover, we reported significant positive correlation between cytokines [IL-6 (*r* = 0.817, *p* < 0.001) and IL-10 (*r* = 0.928, *p* < 0.001)] and anti-HBsAg titre.

**Conclusion:**

Approximately two thirds of adolescents vaccinated at birth lack protective levels of antibodies against hepatitis B virus. Booster vaccines could aid in mounting protective levels of anti-HBsAg. Physical exercise was negatively associated with immune response to hepatitis B vaccinations.

## 1. Introduction

Hepatitis B virus (HBV) is the leading cause of chronic hepatitis, hepatocellular carcinoma, and cirrhosis of the liver. It has infected more than 2 billion people with 350 million individuals having chronic cases worldwide [1]. Chronic hepatitis B is one of the major contributors of liver failure and hepatocellular carcinoma. Cirrhosis or liver cancer kills 15% to 25% of people with chronic HBV infection [2]. Moreover, estimates from the Global Burden of Disease study revealed 686,000 deaths due to hepatitis B in 2013 globally, with 300,000 deaths attributed to liver cancer and 317,400 deaths attributed to cirrhosis of the liver secondary to hepatitis B [2, 3]. This rate represents a significant global burden with a wide geographical variation. The prevalence of chronic HBV infection is highest in Sub-Saharan Africa and the Western Pacific, where countries are classified as high-intermediate to high endemicity (5% to ≥8% prevalence), and prevalence estimates exceed 15% in several countries [2]. Most countries have seen an overall decrease in prevalence of chronic HBV infection over time, with notable increases in African and Eastern European countries [2, 4]. In Ghana, the national prevalence of HBV is 12.3%, which has a significant socioeconomic impact on the country [5].

Numerous preventive measures have been used, including screening blood donors, making plasma-derived products in a way that renders the hepatitis B virus inactive, implementing infection control measures, and giving out hepatitis B immunoglobulin. However, the most significant hepatitis B prevention strategy continues to be active vaccination with the hepatitis B vaccine [6]. In 1992, the WHO directed countries with HBV carrier prevalence of at least 8% to integrate HBV vaccination into their national immunization programs. Thus, HBV-negative babies should be vaccinated against the virus within 24 hours after birth [7, 8]. The aim of this vaccination program is to render many individuals immune to the virus, thereby providing protection from HBV infection. In response to the WHO directive, Ghana initiated mass immunization against hepatitis B infection in children in 2002 in the Expanded Programme on Immunization (EPI).

Nonetheless, concerns have been raised regarding the effectiveness of the vaccines to provoke immune response and long-term protection. Inability of these vaccines to provide protection for vaccinated individuals could lead to new and break-through infections. Moreover, response to vaccination may be diminished by individual characteristics including smoking, obesity, genetic factors, the male sex, route of administration, and immune suppression [9].

Dassah et al. conducted a cross-sectional study in Ghana, assessing hepatitis B vaccine seroconversion in young children aged 0 to 5 years. They reported a 100% seroconversion rate in participants but observed a decline in protective antibody levels over time, suggesting the need for booster doses after five years [6]. However, this observation has not been factored into the immunization program in Ghana, and the study by Dassah et al. remains the sole available data on the EPI program in the Ghanaian population. The current study evaluated seroconversion and seroprotection among HBV-vaccinated adolescent and further assessed factors associated with immune response after booster administration.

## 2. Materials and Methods

### 2.1. Study Design and Study Site

This was a longitudinal cross-sectional study conducted between April 2021 and September 2022. The study was carried out within some selected areas in the Kumasi metropolis. Study participants were recruited from areas including Atonsu, a suburb in Kumasi in the Asokwa Municipal District, Krofrom, and Kwame Nkrumah University of Science and Technology.

### 2.2. Study Population

The study population included 74 adolescents aged 13 to 19 years, who were enrolled in the Expanded Programme on Immunization and were vaccinated against hepatitis B at birth.

### 2.3. Inclusion and Exclusion Criteria

Adolescents aged 13 to 19 years who were fully vaccinated against hepatitis B at birth in the EPI programme (at months 0, 1, and 6) were included in the study, whilst people aged below or above the stated age range regardless of their vaccination status, were excluded.

### 2.4. Ethical Consideration

This study was approved by the Committee for Human Research, Publication, and Ethics (CHRPE) of Kwame Nkrumah University of Science and Technology, with reference number CHRPE/AP/142/21. Written informed consent was obtained from each study participants. Guardians of individuals below 18 years of age consented to their wards' participation in the study.

### 2.5. Sample Collection

Four milliliters (4 ml) of whole blood were taken before and 4 weeks after booster administration from each participant into gel tubes. The blood samples were separated to get serum using a centrifuge (Centurion Scientific, K3 Series) at 3000 rpm for 5 minutes. Serum from each participant was aliquoted into Eppendorf tubes and stored at −80°C, until further processing.

### 2.6. HBV Screening

HBV profile test was performed on each sample using Ovios Rapid Diagnostic Test Kit (Canada). This kit measures HBsAg and HBeAg by a two-site Sandwich method while detection of HBsAb was based on a membrane precoated with HBsAg. HBeAb and HBcAb were measured based on a neutralization competitive inhibition method. Though a qualitative test, it is able to detect HBsAg concentration of ≥2 ng/mL, HBeAg concentration of ≥2 NCU/mL, HBsAb concentration of HBeAb ≥30 mIU/mL, and HBcAb ≥1 : 5. Three drops of serum (approximately 75 *µ*L) were placed in each of the sample wells using Pasteur pipette. Results were read after 15 mins, and HBsAg, HBsAb, and HBeAg were positive if a violet line appeared at both the control and test regions for each of the parameters. However, a violet line at the control region only, with no line at the test region for HBeAb and HBcAb indicates a positive test.

### 2.7. Definition and Classification of HBV Infection

Based on the various HBV serological markers, participants were classified as vaccinated, infected, or recovered.

### 2.8. Measurement Hepatitis B Surface Antibody (HBsAb)

HBsAb was estimated using human ELISA test kit (Shanghai Chemical Ltd. in China). Measurements were done according to the manufacturer's instructions. The kit uses purified antigen to coat microtiter plate wells to make solid phase antigen. Introduction of sample into the well, and subsequent addition of horseradish peroxidase (HRP) enzyme makes an antigen-antibody-enzyme complex. Addition of 3, 3′, 5, 5′-tetramethylbenzidine (TMB) serves as substrate to the HRP. The reaction is stopped by adding sulphuric acid solution and the absorbance/optical density (OD) of intensity of colour developed is read at wavelength of 450 nm. The concentration of HBsAb in the samples is then determined by comparing the OD of the samples to the standards provided on a standard graph.

### 2.9. Classification of Anti-HBsAg Titre

Seroconversion—anti-HBsAg ≥1 mIU/ml. Seroprotection—anti-HBsAg ≥10 mIU/ml. Hyporesponse—10 ≤ anti-HBsAg titre ≤99 mIU/ml.

### 2.10. Booster Immunization

Following the baseline anti-HBsAg estimation, participants with anti-HBsAg titre <10 mIU/ml, were given booster vaccinations of *R*_*x*_ Hepatitis B vaccine (rDNA) I.P (lot number: 0350L001, dosage 20 *µ*g, by Serum Institute of India Pvt. Ltd.). 1 mL of the booster vaccine containing approximately 20 *µ*g of the purified hepatitis B surface antigen was administered intramuscularly into the upper deltoid muscle. Four weeks later, 4 mL of blood were taken for postbooster anti-HBsAb estimations.

### 2.11. Measurement of Cytokines

Cytokines including IL-6 and IL-10 were estimated with an ELISA kit (Shanghai Chemical Ltd., China). The various cytokines were estimated according to the manufacturer's instructions.

### 2.12. Statistical Analysis

Statistical analysis was performed using R programming language (version 4.02). Categorical variables were presented as frequency and percentages, whilst continuous data were presented as mean (±standard deviation) or median (interquartile range) depending on the normality. Association between categorical variables was performed using Pearson's chi-square test or Fisher's exact. Paired sample *T* test was used to compare differences in anti-HBsAg titre before and after booster vaccine administration, whilst the Mann–Whitney *U* test was used to compare differences in immune response between study groups. The one-way ANOVA followed by Tukey's post hoc multiple comparison test was performed to compare differences in immune response between age categories (13–15, 16–18 and >18 years). *p* < 0.05 was considered statistically significant.

## 3. Results

### 3.1. Lifestyle Characteristics of Adolescents Enrolled in the Expanded Programme on Immunization (EPI)

This study enrolled 74 Hepatitis B-vaccinated individuals, of which, majority were within 13–15 years (35.8%), followed by 16–18 years (32.8%) and above 18 years (31.3%). The median body mass index (BMI) was 21.1 kg/m^2^ whilst that of the waist hip ratio was 0.8. The study comprised of 59.5% male participants whilst the remaining 40.5% were females. Considering ethnicity, most of the participants were Akans (85.1%) whilst few were Northerners (1.5%). Moreover, the participants had no history of smoking nor alcohol intake with the majority engaged in exercise activities (61.2%). Over two thirds of the participants had their vaccination at government facilities (95.3%) (Table 1).

### 3.2. Characteristics of Antibody Response to Hepatitis B Virus among Adolescents Who Were Vaccinated in the EPI Programme

Of the 74 adolescents who were vaccinated during the Expanded Programme on Immunization enrolled in the study, 25.7% were seroprotected (had anti-HBsAg levels ≥10 mIU/ml) whilst majority (74.3%) were unprotected (anti-HBsAg levels <10 mIU/ml). The geometric mean titre for age group 13 to 15 was 5.7 (±4.1) mIU/ml, for 16 to 18, 7.3 (±5.8) mIU/ml whilst that of those above 18 years was 18.0 (±16.8) mIU/ml (Table 2). Following seroprotection analysis, only 8.3% of those within the age of 13 to 15 were seroprotected, whilst 22.7% among those within 16 to 18 years were also seroprotected. More than half of those above 18 years were seroprotected.

### 3.3. Sociodemographic Factors Associated with Antibody Response among Adolescents Vaccinated during the EPI Programme

Presented in Table 3 are the lifestyle and clinical factors associated with immune response to hepatitis B vaccination among adolescents enrolled in this study. This study found that age (*p*=0.001) and gender (*p*=0.009) of adolescent were significantly associated with immune response to hepatitis B vaccination. Moreover, exercise was significantly associated with immune response to hepatitis B vaccination (*p*=0.044). However, there was no significant association between ethnicity (*p*=0.436), facility of vaccination (*p*=0.208), and immune response to hepatitis B vaccination (Table 3).

### 3.4. Characteristics of Antibody Response to HBV following Booster Administration

**Figure 1:**
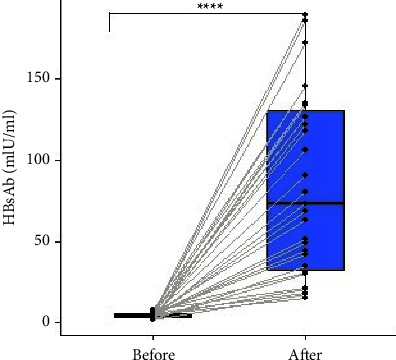
Depicts difference in anti-HBsAb levels before and after HBsAg vaccine booster administration among adolescents who were unprotected in the EPI programme, paired sample *T* tests *p* value is depicted in the plot (^*∗∗∗∗*^ = *p* < 0.0001).

Among the 55 adolescents who were unprotected, 32 agreed to receive the booster doses. The level of anti-HBsAb antibodies was higher after booster shots compared to the levels before the booster doses. Following a paired *T* test analysis, the study found a significant difference between anti-HBsAb antibody levels before and after booster administration (*p* < 0.0001) (Figure 1). Of the 32 participants who were given the booster doses, 40.6% had good response (had anti-HBsAb titre ≥100 mIU/ml) whilst the remaining 59.4% had hyporesponse (had 10 ≤ anti-HBsAb titre ≤99 mIU/ml) to the hepatitis B booster shots.

### 3.5. Difference in Immune Response among Adolescents Who Received Booster Doses

**Figure 2:**
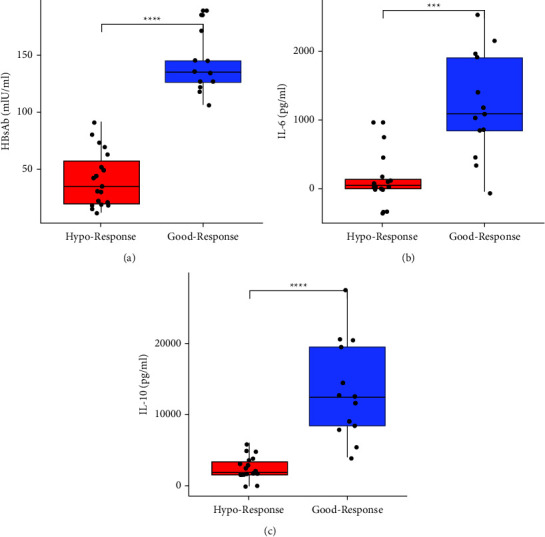
Difference in levels of (a) anti-HBsAb titre, (b) IL-6, and (c) IL-10 between hypo and good responders after vaccine booster administration. Mann–Whitney test *p* value is depicted in the plot, ^*∗∗∗*^ = *p* < 0.001, ^*∗∗∗∗*^ = *p* < 0.0001.

Comparing the immune response after the booster shots, there was a significant difference in anti-HBsAg levels between the hyporesponders and good responders (*p* < 0.0001) (Figure 2(a)). Moreover, the level of cytokine IL-6 was higher among individuals with good response compared to those with hyporesponse (*p* < 0.001). Similarly, the concentration of cytokine IL-10 was also higher in those with good response to the HBsAg vaccine boosters compared to the hyporesponders (*p* < 0.0001) (Figures 2(b) and 2(c)).

### 3.6. Sociodemographic Factors Associated with Anti-HBsAb Response among Adolescents Who Received Booster Doses

Presented in Table 4 are the lifestyle and clinical factors associated with anti-HBsAb production following booster administration. This study found significant association between exercise and anti-HBsAb immune response (*p*=0.034). On the contrary, age category (*p*=0.740), gender (*p*=0.169), ethnicity (*p*=0.523), and facility of vaccination (*p*=0.346) were not significantly associated with immune response after booster administration (Table 4).

### 3.7. Correlation between BMI, WHR, Immune-Mediated Cytokines, and Anti-HBsAg Levels

**Figure 3:**
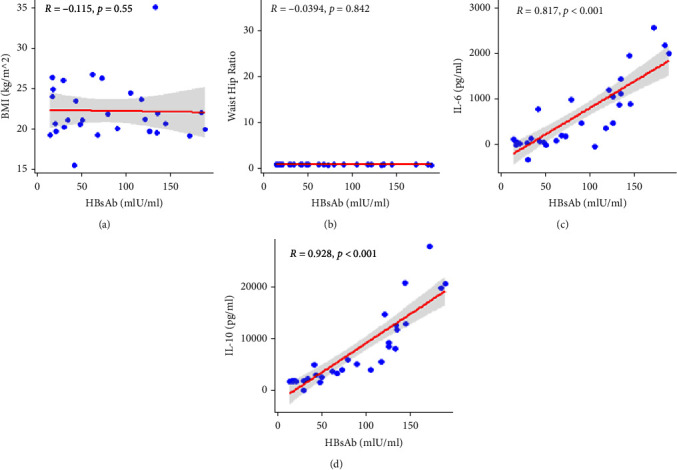
Correlation between anti-HBsAb immune response and (a) Bone marrow index (BMI), (b) Waist hip ratio (WHR), (c) IL-6, and (d) IL-10, *p* > 0.05 was considered statistically insignificant.

In this study, there was insignificant negative correlation among BMI (*r* = −0.115, *p*=0.55), WHR (*r* = −0.0394, *p*=0.842), and serum levels of anti-HBsAb. However, we found significant positive correlations between anti-HBsAg levels and immune-mediated cytokines including IL-6 (*r* = 0.817, *p* < 0.001) and IL-10 (*r* = 0.928, *p* < 0.001) (Figure 3).

## 4. Discussion

The WHO recommends that HBV-negative babies in countries with a prevalence of at least 8% be vaccinated against the virus [8, 10]. Ghana initiated mass immunization against hepatitis B infection in children in 2002 in the Expanded Programme on Immunization (EPI). Nonetheless, concerns have been raised regarding the effectiveness of the vaccines to provoke immune response and long-term protection. Moreover, key challenges to the success of the EPI programme in our part of the world are limited literature or data on immune response among individuals enrolled in this programme. The dearth of data impedes effective actions and decision-making towards the success of the EPI programme in reducing the rate of HBV seroprevalence. In view of this, we evaluated factors associated with immune following HBV vaccination among adolescents who were enrolled in the EPI programme. In this study, there was 100% seroconversion rate, 74.3% had nonprotective antibody levels (anti-HBsAg <10 mIU/ml) whilst 25.7% were seroprotected (anti-HBsAg >10 mIU/ml) after 18 years of vaccination. Gender, age, and exercise were significantly associated with immune response to HBV vaccine. Following booster administration, 59.4% (19/32) had hyporesponse (10 ≤ anti-HBsAb titre ≤99 mIU/ml) whilst 40.6% had good response (anti-HBsAb titre ≥100 mIU/ml) to the HBV booster doses. Exercise was the lifestyle factor associated with immune response after booster shots. Moreover, we observed significant positive correlation between cytokines (IL-6 and IL-10) and anti-HBsAb titre. On the contrary, this study found insignificant negative correlation between BMI, WHR, and antibody levels (anti-HBsAg).

Our findings are consistent and comparable with those of a longitudinal study conducted in Brazil by Freitas da Motta et al., 2002, who reported that 98% of individuals seroconverted [11]. Similarly, in a cross-sectional study in Bangladesh, Chakraborty et al. reported 100% seroconversion rate [12]. Although it has been estimated that those who had received the full course of primary HBV vaccination would be protected for at least 20 years [13], the present study revealed seroprotection rate of 25.7% (anti-HBsAb >10 mIU/ml) among adolescents enrolled in the EPI programme. In contrast, previous studies reported seroprotection rates of 57.7%, 40%, and 74.2% in Egypt, Micronesia, and India, respectively [14–16]. Moreover, seroprotection levels of 86.8% and 90.0% were reported in two separate studies conducted in South Africa and Brazil, respectively [17, 18]. In 2010, Guho and colleagues in Bangladesh also reported 88.67% seroprotection in their study [19]. These high rates of seroprotection were reported among children and infants. Therefore, the lower rate of seroprotection (25.7%) observed among adolescents in our study and others provide additional evidence that antibody concentrations among those who were vaccinated at birth continue to decline, with only about one fifth having protective antibody titers 13–15 years after primary vaccination [14, 20–22].

The current study observed significant associations between age, gender, exercise, and immune response to HBV vaccination. The percentage of male participants that were unprotected was 50.9% whilst unprotection was 49.1% among females. Consistently, a Pakistani study revealed significant association between gender and hepatitis B immune response, with male nonresponders being more than twice that of their female counterparts [23]. It has been postulated that sex hormones influence the epigenetic regulation of genetic expression and gene structure on the X chromosome, resulting in sexual dimorphism in vaccine response [24]. Estrogen stimulates monocytes to secrete IL-10, which in turn stimulates IgG and IgM secretion through B cells, whereas testosterone inhibits the production of IgG and IgM from B-lymphocytes as well as the production of IL-6 from monocytes [9]. This could explain the significant difference in immune response observed between male and female gender. Consistent with the findings in adults, previous studies have reported significant association between age and immune response to HBV vaccination [20, 23]. Although immunity wanes over time, we recorded a higher number of individuals aged over 18 years being seroprotected compared to those below 18 years. This could be due to the fact that, those above 18 years have been exposed to the hepatitis B virus and as such developed natural immunity contributing to seroprotection. However, this had not been confirmed by anti-HBc.

In this current study, exercise was negatively associated with HBV immune response before and after vaccine booster administration. Some studies have also revealed negative effect of exercise on immune response, which is consistent with our finding [25, 26]. Similarly, a study by Bruunsgaard and colleagues demonstrated that exercise disrupted the development of vaccine antibodies [27]. Contrarily, previous reports have revealed a significant positive association between physical activity or fitness and antibody responses to vaccinations in a number of cross-sectional studies of older adult populations [28–30]. Moreover, regular exercise has been linked to improved immune responses to vaccination, decreased numbers of exhausted/senescent T cells, increased T cell proliferative capacity, decreased levels of inflammatory cytokines in the blood (also known as “inflamm-aging”), increased neutrophil phagocytic activity, lowered inflammatory response to bacterial challenge, increased NK-cell cytotoxic activity, and longer leukocyte telomere lengths in aging humans [31]. The adverse effect of exercise on immune response has been attributed to prolonged or strenuous exercise [25, 27]. Many researchers believe that repeated bouts of strenuous exercise lasting over two hours can compromise the immune system [32]. It has been postulated that prolonged or strenuous exercise may suppress the immune system, leading to decreased production of antibodies in response to the vaccine [27]. Although the intensity of exercise was not determined in the present study, this phenomenon could explain why higher percentage of adolescents who exercised were unprotected compared to those who did not exercise.

A significant increase in surface antibody levels was observed 4 weeks following booster vaccination, with 100% of boosted participants having detectable antibody levels. However, 59.4% had a poor response (hyporesponse) to booster vaccines. Given that a prior study evaluating immune response to HBV vaccines in Ghana, reported 32.61% nonresponse, it is possible that these individuals were primary non responders [20]. Conversely, some presumed nonresponders may actually be slow responders, which would explain their low rate of memory accumulation [33, 34].

However, following booster shots, there was insignificant negative correlation between BMI, WHR, and anti-HBsAg levels. This finding is inconsistent with a study by Middleman et al., which reported that higher BMI was significantly associated with lower rates of seroprotection [35]. Similarly, Kar et al. also found that higher BMI was significantly associated with nonprotective levels of antibodies [15]. The disparity in results could be attributed to our small sample size. Although insignificant negative correlation was observed between BMI and antibody levels, the negative correlation could be due to hormonal influences present in participants with high BMI that may affect immune response [36]. Alternatively, it is possible that the vaccine was not fully injected into muscle but rather into subcutaneous fat, which would have decreased immune response as hypothesized by previous study [35, 36]. Also, low response of overweight people to vaccines could be attributed to the vaccine's primary distribution in fat rather than muscle [37, 38]. This could obstruct absorption and allow enzymatic denaturation of the vaccine antigen.

Th1 cytokines such as IL-2, IFN-, and transforming growth factor beta (TGF-) induce a cell-mediated immune response that leads to the cure or destruction of HBV-infected hepatocytes [39]. Whereas Th2 cytokines such as IL-6, IL-10, and IL-13 stimulate humoral immune response, which is required for B cell differentiation and specific antibody production, which is responsible for virus clearance [39, 40]. The current study revealed significant increased levels of both cytokines IL-6 and IL-10 among those who were good responders to the hepatitis B vaccine boosters, with a significant positive correlation between these cytokines (IL-6 and IL-10) and anti-HBsAb titre. Consistent with our findings, Velu et al. reported that Th2 cytokines including IL-10 and IL-4, were secreted at significantly higher levels in high-responders compared with hyporesponders and nonresponders [41]. The process of producing antibodies against this HBsAg is T cell-dependent and requires Th cell activation. IL-6 has long been known to stimulate B cell differentiation and is also a major inducer of antibody production [42]. The higher levels of IL-10 observed in good responders could be attributed to its potential capacity to enhance antibody production through a Th2-type response and B cell maturation [43]. Moreover, its anti-inflammatory properties enable the modulation of the inflammatory response to the vaccine [44]. This balanced and controlled reaction facilitates the generation of protective antibodies while mitigating excessive inflammation or tissue damage. Contrarily, the low levels of cytokines IL-6 and IL-10 observed among our study participants with hyporesponse to booster vaccines could be due to a flaw in the primary HBsAg-specific T cell repertoire or antigen presentation [41, 45].

Although our study is among one of the few studies contributing to literature on hepatitis B vaccination at birth in Ghana, one major limitation is its relatively small sample size. We therefore recommend that future studies incorporate large sample size to effectively study the factors associated with immune response to hepatitis B vaccination.

## 5. Conclusion

Approximately 3 in 4 of adolescents vaccinated at birth in the EPI program lack protective levels of antibodies against Hepatitis B virus after about 18 years of immunization. Administering booster vaccines could aid in mounting protective levels of antibodies against hepatitis B virus. Moreover, high levels of IL-6 and IL-10 are associated with high anti-HBsAg titers. IL-6 stimulates B cell differentiation and antibody production, while higher IL-10 levels in good responders enhance antibody production through a Th2-type response and B cell maturation, contributing to the efficacy of the booster vaccines. Stakeholders should devise strategies geared towards promoting hepatitis booster vaccines coverage.

## Figures and Tables

**Table 1:** Sociodemographic characteristics of study participants.

Variable	Frequency (*n* = 74)	Percentage (%)
Age category^*∗*^		
13–15	24	35.8
16–18	22	32.8
>18	21	31.3
Gender		
Male	44	59.5
Female	30	40.5
Ethnicity^*∗*^		
Akan	57	85.1
Northerner	1	1.5
Ga/Adangbe	9	13.4
Exercise^*∗*^		
Yes	41	61.2
No	26	38.8
Facility of vaccination^*∗*^		
Government	61	95.3
Private	3	4.7
BMI	21.1 (15.46–35.02)	
WHR	0.8 (0.72–0.92)	

Data are presented as frequency (%) or median (IQR), BMI = bone marrow index, WHR = waist hip ratio, ^*∗*^indicates variable with missing values.

**Table 2:** Antibody titre among the various age groups.

Age group (years)	Total (%)	Mean titre (mIU/ml) (95%, CI)	*p* value
13–15	24 (35.8)	5.7^b*∗*^ (3.9–7.4)	<0.001
16–18	22 (32.8)	7.3^ba^ (4.7–9.8)	
>18	21 (31.3)	18.0^a*∗*^ (10.3–25.7)	

One-way ANOVA followed by Tukey post hoc multiple comparison was performed. ^*∗*^indicates significant difference between 13 and 15 and >18, ^a^ indicates significant difference between 16 and 18 and >18 whilst, ^b^ indicates no statistically significant difference between 13–15 and 16–18.

**Table 3:** Sociodemographic and lifestyle factors associated with antibody response among adolescents enrolled in the EPI programme.

Variable	Immune response		*p* value
	Unprotected (*n* = 55)	Seroprotected (*n* = 19)	
Age category			**0.001**
13–15	22 (45.8)	2 (10.5)	
16–18	17 (35.4)	5 (26.3)	
>18	9 (18.8)	12 (63.2)	
Gender			**0.009**
Male	28 (50.9)	16 (84.2)	
Female	27 (49.1)	3 (15.8)	
Ethnicity			0.436
Akan	42 (87.5)	15 (78.9)	
Northerner	1 (2.1)	0 (0.0)	
Ga/Adanbge	5 (10.4)	4 (21.1)	
Exercise			**0.044**
Yes	33 (68.8)	8 (42.1)	
No	15 (31.3)	11 (57.9)	
Facility of vaccination			0.208
Government	44 (97.8)	17 (89.5)	
Private	1 (2.2)	2 (10.5)	

Data are presented as frequency (%), Chi square/Fisher's exact test, *p* value <0.05, and bolded means statistically significant.

**Table 4:** Sociodemographic factors associated with anti-HBsAb response after vaccine booster administration.

Variable	Immune response		*p* value
	Hyporesponse (*n* = 19)	Good response (*n* = 13)	
Age category			0.740
13–15	6 (35.3)	3 (30.0)	
16–18	6 (35.3)	5 (50.0)	
>18	5 (29.4)	2 (20.0)	
Gender			0.169
Male	7 (36.8)	8 (61.5)	
Female	12 (63.2)	5 (38.5)	
Ethnicity			0.523
Akan	14 (82.4)	9 (90.0)	
Ga/Adanbge	3 (17.6)	1 (10.0)	
Exercise			**0.034**
Yes	14 (82.4)	4 (40.0)	
No	3 (17.6)	6 (60.0)	
Facility of vaccination			0.346
Government	17 (100.0)	8 (88.9)	
Private	0 (0.0)	1 (11.1)	

Data is presented as frequency (%), Chi square/Fisher's exact test, *p* value <0.05 and bolded means statistically significant.

## Data Availability

All data generated or analyzed during this study are included in this article and are available from the corresponding author upon reasonable request.
